# MoLEP vs. HoLEP for BPH: A 3-Year Greek Single-Center Retrospective Comparative Cohort Study on 1368 Cases

**DOI:** 10.3390/cancers17101608

**Published:** 2025-05-10

**Authors:** Panayiotis Veveloyiannis, Nikolaos Bafaloukas, Dimitra S. Mouliou

**Affiliations:** 1 Minimal Invasive Urology Clinic MITERA Hospital HHG Group, 15123 Marousi, Greece; veveloyiannis.panayiotis@gmail.com; 2 Forth Urological Department, IASO Hospital, 15123 Marousi, Greece; bafaloukasn@yahoo.co.uk; 3 Independent Researcher, 38500 Volos, Greece

**Keywords:** HoLEP, MoLEP, benign prostatic hyperplasia, Qmax, PVR, PSA, IPSS, QoL, enucleation time, complications

## Abstract

Background: Holmium Laser Enucleation of the Prostate (HoLEP) is an established treatment for benign prostatic hyperplasia (BPH). Pulse-modulated lasers, like MOSES technology (MoLEP), may enhance the procedure’s efficiency and safety. Methods: A 3-year single-center retrospective comparative study was conducted on 1368 patients treated with HoLEP/MoLEP at MITERA Hospital. Results: A total of 688 patients were treated with HoLEP and 680 with MoLEP. Compared to HoLEP, MoLEP demonstrated shorter surgical (50.5 min [IQR 33–60] vs. 58 min [IQR 46–69], *p* < 0.01) and enucleation times (34 min [IQR 23–43] vs. 43 min [IQR 34–51], *p* < 0.001) and shorter hospital stay (8 h [IQR 6–19] vs. 12 h [IQR 9–24], *p* = 0.027), catheterization time (19 h [IQR 12–48] vs. 24 h [IQR 24–48], *p* < 0.001), and irrigation duration (5 h [IQR 2–8] vs. 7 h [IQR 3–10], *p* < 0.001), with similar morcellated tissue weight and morcellation time. At 1 month, MoLEP showed higher Qmax (27.3 mL/s [IQR 23.9–30.3] vs. 20 mL/s [IQR 17–23.6], *p* < 0.001), lower PVR (11.4 mL [IQR 7.7–15] vs. 12.5 mL [IQR 7–18], *p* = 0.005), better IPSS (4 [IQR 3–6] vs. 7 [IQR 5–11], *p* < 0.005), QoL (1 [IQR 1–2] vs. 2 [IQR 1–2], *p* < 0.001), lower PSA (1.8 ng/mL [IQR 1.1–2.6] vs. 2.4 ng/mL [IQR 1.3–3.5], *p* < 0.001), which were maintained at 6 months, and fewer Clavien-Dindo I (2.5% vs. 7.5%, *p* < 0.001) and II (16% vs. 25.7%, *p* < 0.001) complications. Conclusions: MoLEP offered significant advantages over HoLEP in this study.

## Simple Summary

This study compared HoLEP and MoLEP in 1368 patients with benign prostatic hyperplasia. MoLEP showed significant improvements in surgical efficiency, shorter hospital stays, and reduced complication rates. At 1 and 6 months postoperatively, MoLEP achieved better functional outcomes, including higher Qmax, lower PVR, improved IPSS, and reduced PSA levels. Fewer complications were reported with MoLEP (Clavien-Dindo I and II), suggesting it might be a more favorable option than HoLEP for BPH treatment.

## 1. Introduction

Benign Prostatic Hyperplasia (BPH) is a prevalent condition among men, with its occurrence increasing over time and affecting not only older men, but also younger men [1]. Over the past few years, significant advancements have been achieved in the surgical management of BPH, which have overtaken the traditional surgical techniques.

The introduction of advanced laser techniques, such as Holmium Laser Enucleation of the Prostate (HoLEP), has revolutionized prostate surgery. Due to its effectiveness, consistency, safety, and suitability for all prostate sizes, endoscopic enucleation of the prostate is now becoming the preferred gold standard for surgical treatment [2,3]. HoLEP is now strongly endorsed by both the American Urology Association (AUA) and the European Association of Urology (EAU) and a recent meta-analysis has confirmed its benefits for treating BPH, offering superior perioperative outcomes, faster recovery, and reduced postoperative risks, whereas en Bloc HoLEP is an advanced technique that allows for the complete removal of the prostatic adenoma in a single piece, improving efficiency and preserving the surgical plane [4,5,6,7].

Moses Laser Enucleation of the Prostate (MoLEP), an advanced laser technique, utilizes MOSES^®^ Technology, a pulse modulation method developed by Lumenis that enhances surgical efficiency through the Moses effect [8]. A meta-analysis found that MoLEP demonstrates superior intraoperative efficiency compared to standard HoLEP, with shorter surgical times and reduced length of stay, making it a promising option for same-day prostate surgery; even if early evidence on MoLEP seems promising, more and high-volume studies are needed to reveal its comparative advantages over other enucleation techniques [8,9].

This is the first Greek study to compare HoLEP and MoLEP for the management of BPH, based on a retrospective analysis of 1368 patient cases from MITERA Hospital—the largest survey of its kind in the existing literature. The study aims to provide a comparative evaluation of these surgical techniques by analyzing certain preoperative, intraoperative, and postoperative outcomes. Additionally, it assesses key operational parameters related to their efficacy and performance as well.

## 2. Materials and Methods

### 2.1. The Study Concept and Period

**Figure 1:**
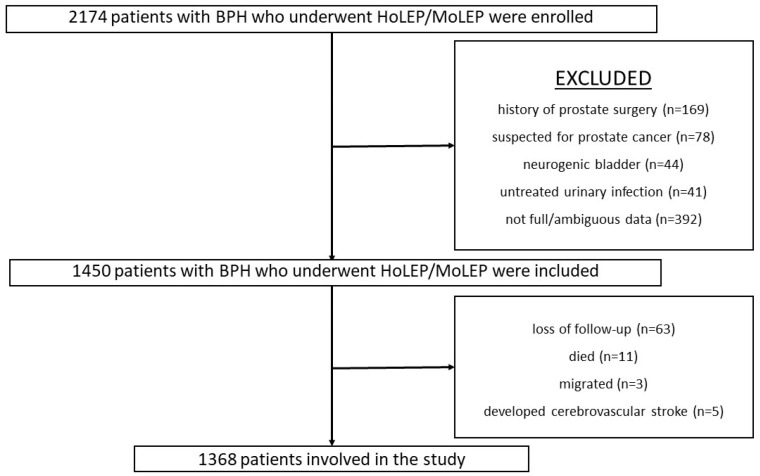
The inclusion and the exclusion flow-chart of the study.

This high-volume single-center retrospective comparative cohort study is based on data collected between May 2022 and March 2025 at MITERA Hospital. The study includes preoperative assessments, surgical procedures, and postoperative follow-ups conducted at 1 and 6 months, respectively. The study included all living male patients who underwent BPH surgery via HoLEP and MoLEP during the aforementioned period, but initially excluded with suspected prostate cancer, partial/ambiguous data, previous prostate/lower urinary tract surgery, untreated urinary infection, and neurogenic bladder, and excluded cases who were lost to follow-up, deceased, or migrated and those who developed a cerebrovascular stroke. Among a total of 2174 patients, 681 (340 HoLEP and 341 MoLEP) surgeries were performed in the first year, 784 (392 HoLEP and 392 MoLEP) in the following year, and 729 (464 HoLEP and 465 MoLEP) in the final year (Figure 1).

### 2.2. The Equipment and the Operation Technique

The HoLEP surgeries were carried out via a 26Fr Storz laser scope, utilizing a Dornier Laser 140 W generator (by Jena) in long-pulse mode with a 550 μm fiber and operating at 120 W derived from 3 Joules × 40 Hz and a Piranha Morcellator, whereas the MoLEP surgeries were performed via the same scope using the Moses 2.0 Laser generator made by Boston along with a 550 micron fiber in Moses pulse mode and 100 W - 2 J - 50 Hz, together with the same Piranha Morcellator. All the procedures were performed with the En Bloc technique and with early sphincter release and at the completion of each procedure, a 22Fr double lumen catheter was inserted into the bladder and all cases passed the TWOC after removal of the catheter.

The surgeries were alternately performed by two highly experienced surgeons, each rotating between HoLEP and MoLEP procedures, both of whom were exclusively trained in HoLEP and MoLEP techniques and had performed over 200 surgeries with the standard en bloc technique.

The same en bloc technique with early sphincter release was used in every procedure regardless of the laser used. This en bloc approach started at 12 o’clock apical part of the adenoma, lowering the mucosa incision vertically down each side of the veru; thus, a mucosa detachment achieved early shielding of the sphincter, protecting it from thermal injury and tearing forces. Then, the enucleating plane was developed symmetrically until adenoma detachment at the bladder neck.

### 2.3. The Measures That Were Analyzed for the Study

Excluding common demographic information, including age, retirement and city of living, and history of COVID-19 infection, this study used data on height and weight to calculate the participants’ BMI, smoking history, diabetes, hypertension, cardiovascular conditions, cancer, as well as some other evidence on general HoLEP indicators such as LUTS, refractory retention, medical treatment failure, obstructive Acute Kidney Disease (AKI), hematuria, bladder stone, recurrent prostatitis, and the possible anticoagulant therapy before the operation.

For the preoperative and the postoperative evaluations, the peak urinary flow rate (Qmax), the Post-Void Residual (PVR), the International Prostate Symptom Score (IPSS) and the QoL score [10,11]; Qmax was measured with uroflowmetry, whereas PVR and prostate volume were assessed with ultrasound, and for those fitted with an indwelling catheter before the surgery, we included only those who could provide full data about such parameters just before they started wearing the catheter. Hemoglobin, hematocrit and serum creatinine were recorded, and also, possible complications and need for blood transfusion were recorded. In addition, intraoperative measures included the morcellated tissue weight, the enucleation time and the morcellation time as well.

### 2.4. Statistical Analysis of the Study

Statistical analyses were performed via SPSS statistics for Windows, Version 29.0 (IBM Corp., Armonk, NY, USA). Tests were two-tailed, and the level of statistical significance was set at *p* ≤ 0.05. To assess the normality of continuous variables, the Shapiro–Wilk test was used. Normally distributed data are presented as mean ± Standard Deviation (SD), while non-normally distributed data are presented as median and InterQuartile Range (IQR; IQR). Data were well balanced; therefore, a Two Independent Samples test and an Independent Samples *t*-test were used for estimates of mean ranks and the Mann–Whitney-U test and Kruskal–Wallis-H test were used to assess medians among subgroups, whereas Pearson and Spearman coefficients were used to evaluate correlations between them. The Chi-square test was applied for comparisons of frequencies.

### 2.5. The Ethical Considerations—Approval of the Study

The study was approved by the Ethics Committee of MITERA Hospital. Informed consent was obtained from all patients involved in this study, with all participants specifically agreeing to take part in the research conducted exclusively by Dr. Veveloyiannis.

## 3. Results

### 3.1. Demographic and Other Information for the Population-Based Sample

This study included 1368 Greek male patients who underwent HoLEP/MoLEP surgery with a median age of 70 (IQR = 9, min = 42, max = 95), of whom 67.1% were from Athens and the 64.8% were external tertiary referrals to MITERA Hospital. Table 1 provides further information on the medical history and Table 2 provides further information on indications for HoLEP/MoLEP surgery, for the population-based sample of this study.

### 3.2. Preoperative Information for the Population-Based Sample

Table 3 provides preoperative information for the population-based sample.

### 3.3. Perioperative Information for the Population-Based Sample

Interestingly, regarding the operational parameters, surgical performance and in-hospital hours of stay, MoLEP showed improved parameters, as seen in Table 4.

### 3.4. Postoperative Information for the Population-Based Sample

All participants who were undergoing anticoagulant/antiplatelet therapy continued it after surgery. Table 5 provides the postoperative information for the population-based sample.

No patient required a blood transfusion, whereas only five cases who were treated with HoLEP required a second surgery, and this was performed about 3 months after their first surgery.

## 4. Discussion

This is the first high-volume real-world single-center retrospective comparative cohort study on HoLEP and MoLEP, and also this is the first Greek study on this topic. Participants tended to follow the known Greek epidemiological rates concerning the various underlying medical conditions, which sometimes may trigger failure of medical therapies for other conditions, whereas, in general, it has been discussed that systemic inflammation can have a negative impact on various therapies [12,13,14,15]. In this study, the general indications for surgery were similar between HoLEP and MoLEP, with no significant differences in the rates of LUTS, refractory retention, medical treatment failure, obstructive AKI, persistent hematuria, bladder stones, or recurrent prostatitis/urinary infections. Preoperative parameters, including prostate volume, Qmax, PVR, IPSS, QoL, PSA levels, hemoglobin, hematocrit, and serum creatinine, were also comparable between the two groups. However, during the perioperative period, MoLEP demonstrated shorter surgical and enucleation times (50.5 vs. 58 min, *p* < 0.01; 34 vs. 43 min, *p* < 0.001, respectively), reduced hospital stay (8 h vs. 12 h, *p* = 0.027), shorter catheterization time (19 h vs. 24 h, *p* < 0.001), and less irrigation duration (5 h vs. 7 h, *p* < 0.001), with no significant differences in morcellated tissue weight or morcellation time. Postoperatively, MoLEP outperformed HoLEP in functional outcomes, showing higher Qmax (27.3 vs. 20 mL/s, *p* < 0.001), lower PVR (11.4 vs. 12.5 mL, *p* = 0.005), better IPSS (4 vs. 7, *p* < 0.005), improved QoL (1 vs. 2, *p* < 0.001), and reduced PSA levels (1.8 ng/mL vs. 2.4 ng/mL, *p* < 0.001). Additionally, MoLEP was associated with fewer Clavien-Dindo I (2.5% vs. 7.5%, *p* < 0.001) and II (16% vs. 25.7%, *p* < 0.001) complications at one month. At six months, MoLEP continued to show superior outcomes in terms of Qmax, IPSS, QoL, and PSA levels. Surgical complications at six months were also lower in the MoLEP group, with a significantly lower rate of Clavien-Dindo I complications (0.7% vs. 0.1%, *p* = 0.035). These findings suggest that MoLEP offers improved functional outcomes and fewer complications compared to HoLEP, both in the short and long term. In particular, the lower incidence of incontinence in the MoLEP group may be attributed to MoLEP’s more precise and less invasive technique, which causes less damage to surrounding tissues and muscles compared to HoLEP, along with the use of MOSES technology, which allows for more accurate tissue removal.

In our study, we found a statistically significant difference for MoLEP in terms of surgical and enucleation times, as well as for the length of hospital stay after surgery, catheterization, and irrigation times as well (Table 4). Socarrás et al. [16] compared en bloc HoLEP and en bloc MoLEP, again in a small sample size, and reported a better enucleation time for MoLEP, and also, the hemostasis time, ablation rate and the overall surgical time were significant in those managed with MoLEP. Also, the study of Tong et al. [17], which was again based on a small sample size, found a shorter enucleation time and hemostasis time for those who underwent MoLEP, along with a superior enucleation efficiency. It should be noted that the first HoLEP studies reported significantly higher times, especially the enucleation time and the morcellation time, as seen in a study by Monn et al. [18] that was published a decade ago. Apart from that, a relatively recent review of seven studies on MoLEP technology concluded that, compared to standard HoLEP, the mean PVR, mean enucleation time and postoperative length of stay were shorter, but Qmax was not significantly improved; however, in our study, we found an improvement in Qmax, a fact which is in parallel with several other studies [8]. A review on MoLEP has highlighted a preservation of HoLEP objective and subjective outcomes as well, but it has also noted successful same-day catheter removal and same-day discharge in various patients [9].

For over a decade, HoLEP has been recognized as a safe and effective treatment option for patients on anticoagulants with symptomatic BPH that does not respond to medical therapy. As seen in Table 5, we found that one month and six months after surgery, MoLEP was superior to HoLEP for Qmax, IPSS, QoL, PSA, and post-surgical complications, and also only few HoLEP cases required a second surgery. Banga-Mouss et al. [19] also reported that HoLEP significantly improved the Qmax compared to its preoperative value, yet we found better values and also postoperative hematuria, which was present at 1/3 in their patients, was significantly lower in our study, but as was the case in their study, complications were more likely to be recorded soon after the introduction of HoLEP. Actually, compared to traditional methods, Qmax has been found to be improved in HoLEP, except in one study. The first prospective randomized trial in 140 patients who underwent HoLEP/MoLEP showed that no statistically significant difference for PSA and mean prostate volume was recorded in HoLEP versus MoLEP-managed patients, yet this study had a low volume [20]. Yet, one should bear in mind that while MoLEP demonstrates superior outcomes in terms of surgical efficiency and functional recovery, HoLEP remains a valuable option due to its long-established safety profile, proven efficacy, and widespread familiarity in clinical practice. Additionally, MoLEP’s newer technology may not be as universally accessible or affordable, making HoLEP a continued first-line choice for many healthcare settings.

The basic strength of this real-world study is that it includes a large sample size of 1368 patients and it spans 3 years, providing longitudinal data on preoperative and postoperative outcomes and allowing for an assessment of both short-term and long-term impacts. Additionally, by including both HoLEP and MoLEP procedures, this research offers comparative insights into two advanced minimally invasive techniques, which are some modern urological practices. Also, the inclusion of all demographic, clinical, and procedural variables ensures a holistic understanding of the outcomes. As a result, this study adds valuable information on the implementation of minimally invasive techniques in Greece, with implications for global healthcare practices. Finally, such a high-volume retrospective study may be better than a randomized controlled trial because it reflects real-world clinical practice, includes a broader patient population, and provides more generalizable results, whereas the last one operates under highly controlled conditions with strict inclusion criteria that may limit external validity; besides, this study was naturally well balanced without the need for additional adjustments that could artificially alter people’s characteristics and potentially mask some real clinical variations.

However, since, in reality, all studies have some limitations, firstly, the exclusion of certain cases could have a negative impact on the generalizability of the findings. Also, the reliance on self-reported information may introduce inaccuracies or incomplete information.

## 5. Conclusions

This high-volume real-world single-center retrospective comparative cohort study highlights key differences between HoLEP and MoLEP, with MoLEP offering better postoperative outcomes and procedural efficiency. Patients treated with MoLEP experienced greater improvements in urinary flow, symptom relief, and quality of life, with fewer complications like infections and bleeding. MoLEP also had shorter surgical and recovery times. MoLEP was identified as a faster and more effective method, providing better quality of life after surgery. These findings suggest that MoLEP is a great approach for managing BPH, especially for patients requiring quicker recovery and fewer complications.

## Figures and Tables

**Table 1:** General information on the medical history of the population-based sample.

	HoLEP N_1_ = 688	MoLEP N_2_ = 680	*p*-Value
General Information			
Age (median, IQR, min-max)	71 (77–64, 42–95)	70 (74–67, 42–94)	0.629
BMI (median, IQR, min-max)	24.2 (28.2–20.3, 16.7, 39.1)	24.1 (27.3–21.4, 16.9, 38.7)	0.241
Smoking (n, % out of N)	253 (36.8)	251 (36.9)	0.958
Diabetes (n, % out of N)	107 (15.6)	117 (17.2)	0.409
Hypertension (n, % out of N)	364 (48.9)	380 (51.1)	0.269
Cardiovascular disease (n, % out of N)	80 (11.6)	83 (12.2)	0.741
Respiratory disease (n, % out of N)	62 (9)	74 (10.9)	0.248
Neurologic disease (n, % out of N)	63 (9.2)	68 (10)	0.596
Immunosuppressed (n, % out of N) ^1^	109 (15.8)	115 (16.9)	0.593
History of cancer (n, % out of N)	34 (4.9)	48 (7.1)	0.099
Anticoagulant/antiplatelet therapy (n, % out of N) ^2^	244 (49.5)	249 (50.5)	0.657

^1^ Immunosuppressed patients include those undergoing chemotherapy and other immunosuppressive medication; ^2^ about 1/3 of HoLEP and 1/3 of MoLEP were on oral anticoagulants and the others were undergoing antiplatelet therapies.

**Table 2:** General indications for HoLEP/MoLEP surgery for the population-based sample.

	HoLEP n (% Out of N_1_) N_1_ = 688	MoLEP n (% Out of N_2_) N_2_ = 680	*p*-Value
General Indications for HoLEP/ MoLEP Surgery			
LUTS	535 (77.8)	521 (76.6)	0.614
Refractory retention	51 (7.4)	58 (8.5)	0.446
Medical treatment failure	257 (37.4)	261 (38.4)	0.695
Obstructive AKI	74 (10.8)	80 (11.8)	0.555
Persistent hematuria	38 (5.5)	46 (6.8)	0.339
Bladder stone	203 (29.5)	211 (31)	0.540
Recurrent prostatitis/urinary infections	83 (12.1)	79 (11.6)	0.798

**Table 3:** Comparison of HoLEP and MoLEP based on the preoperative information of the population-based sample.

	HoLEP	MoLEP	*p*-Value
Preoperative Information			
Prostate volume (cc) (median, IQR, min-max)	123 (97–152, 39–396)	127 (103–160, 40–398)	0.062
Qmax (mL/s) (median, IQR, min-max)	7.3 (3.2–11.5, 1–24.7)	7 (3–11.5, 1–24.1)	0.333
PVR (mL) (median, IQR, min-max)	141 (94–209, 11, 589)	143 (98–187, 13, 601)	0.521
IPSS (median, IQR, min-max)	24 (20–29, 5–35)	24.3 (19–30, 5–35)	0.463
QoL (median, IQR, min-max)	5 (4–6, 6–2)	5 (4–6, 6–1)	0.880
PSA (ng/mL) (median, IQR, min-max)	9.56 (6.7–12.2, 0.45–17.45)	9.5 (7.2–11.5, 0.64–18.67)	0.492
Hemoglobin (gr/dL) (median, IQR, min-max)	14.7 (13.6–16, 8.3–18.5)	14.8 (13.6–16, 8.2–18.6)	0.820
Hematocrit (%) (median, IQR, min-max)	45.3 (42.3–49.4, 25.5–56.8)	45.7 (42.3–49.4, 27.1–57.1)	0.830
Serum creatinine (μmol/L) (median, IQR, min-max)	86.7 (70.9–105.3, 27.3–156.5)	86.9 (73.3–107.2, 23.4–158.3)	0.117

**Table 4:** Comparison of HoLEP and MoLEP based on perioperative information and operational performance in the population-based sample, as well as time in hospital, irrigation time, and catheterization time.

Operational Parameters and Performance	HoLEP	MoLEP	*p*-Value
Morcellated tissue weight (gr) (median, IQR, min-max)	88.9 (70.4–110.6, 29.5–325.6)	89.2 (72–112.2, 26.2–292.4)	0.351
Surgical time (min) (median, IQR, min-max)	58 (46–69, 17–110)	50.5 (33–60, 14–115)	<0.01
Enucleation time (min) (median, IQR, min-max)	43 (34–51, 14–78)	34 (23–43, 11–80)	<0.001
Morcellation time (min) (median, IQR, min-max)	16.5 (15.6–17.8, 3–32)	16.6 (15–18.1, 3–37)	0.118
Hours of stay in hospital (h) (median, IQR, min-max)	12 (9–24, 7–24)	8 (6–19, 6–24)	0.027
Catheterization time (h) (median, IQR, min-max)	24 (24–48, 10–48)	19 (12–48, 8–48)	<0.001
Irrigation duration (h) (median, IQR, min-max)	7 (3–10, 1–16)	5 (2–8, 1–15)	<0.001

**Table 5:** Comparison of HoLEP and MoLEP based on the postoperative information of the population-based sample.

	HoLEP	MoLEP	*p*-Value
1-Month Postoperative Information			
Prostate volume (cc) (median, IQR, min-max)	34 (27–42, 9–78)	36 (28–45, 13–106)	0.089
Qmax (mL/s) (median, IQR, min-max)	20 (17–23.6, 13,1–35.5)	27.3 (23.9–30.3, 14.6–37.8)	<0.001
PVR (mL) (median, IQR, min-max)	12.5 (7–18, 0–90)	11.4 (7.7–15, 1–61)	0.005
IPSS (median, IQR, min-max)	7 (5–11, 0–30)	4 (3–6, 0–24)	<0.005
QoL (median, IQR, min-max)	2 (1–2, 0–4)	1 (1–2, 0–3)	<0.001
PSA (ng/mL) (median, IQR, min-max)	2.4 (1.3–3.5, 0.13–8.52)	1.8 (1.1–2.6, 0.09–5.13)	<0.001
Hemoglobin (gr/dL) (median, IQR, min-max)	14.1 (12.8–15.7, 8–18.4)	14 (12.2–15.9, 7.3–18.6)	0.868
Hematocrit (%) (median, IQR, min-max)	43.4 (39.3–46.9, 25.6–56.6)	43.5 (39.5–49.6, 24–56)	0.266
Serum creatinine (μmol/L) (median, IQR, min-max)	82.6 (70.1–96.8, 47.9–122.5)	82.5 (71–95.3, 16.3–223.5)	0.732
**Surgical Complications reported at the 1st month (Clavien-Dindo Classification)**			
Clavien-Dindo I (n, % out of N)	50 (7.5)	17 (2.5)	<0.001
*Urinary incontinence (stress)*	*36 (5.2)*	*17 (2.5)*	*0.008*
*Hematuria*	*14 (2.3)*	*0 (0)*	*-*
Clavien-Dindo II (n, % out of N)	177 (25.7)	109 (16)	<0.001
*Hematuria*	*69 (10)*	*36 (4.4)*	*0.001*
*Urinary infections*	*108 (15.7)*	*73 (12.2)*	*0.006*
Clavien-Dindo III-IV (n, % out of N)	4 (0.6)	0 (0)	-
**6-Month Postoperative Information**			
Prostate volume (cc) (median, IQR, min-max)	35 (28–43, 13–57)	37 (29–44, 12–89)	0.217
Qmax (mL/s) (median, IQR, min-max)	20.6 (17.6–23.4, 9.5–39.5)	28.6 (26–31.3, 16.8–40.3)	<0.001
PVR (mL) (median, IQR, min-max)	7 (3–11, 0–33)	6.8 (3.7–10, 0–25.4)	0.124
IPSS (median, IQR, min-max)	3 (2–3, 0–9)	2 (1–2, 0–5)	<0.001
QoL (median, IQR, min-max)	1 (1–2, 0–2)	0 (0-0, 0–1)	<0.001
PSA (ng/mL) (median, IQR, min-max)	1.32 (0.79–1.8, 0.07–3.21)	1.06 (0.9–1.2, 0.44–1.65)	<0.001
Hemoglobin (gr/dL) (median, IQR, min-max)	14.3 (13.1–15.6, 8.2–18.2)	14.5 (13.5–15.5, 8.1–19.4)	0.118
Hematocrit (%) (median, IQR, min-max)	44.6 (40.8–48.4, 25.6–56.5)	44.7 (40.9–48.5, 24.8–56.2)	0.962
Serum creatinine (μmol/L) (median, IQR, min-max)	90.1 (82.1–99.1, 50.2–124.9)	90.3 (73.9–106.4, 21.7–129)	0.428
**Surgical Complications reported at the 6th month (Clavien-Dindo Classification)**			
Clavien-Dindo I (n, % out of N)	7 (0.7)	1 (0.1)	0.035
Clavien-Dindo II (n, % out of N)	-	-	-
Clavien-Dindo III–IV (n, % out of N)	-	-	-

## Data Availability

Data sharing is not applicable to this article. The data are not publicly available due to restrictions. They contain information that could compromise the privacy of the participants.
